# Mutual Information-Driven Feature Reduction for Hyperspectral Image Classification

**DOI:** 10.3390/s23020657

**Published:** 2023-01-06

**Authors:** Md Rashedul Islam, Boshir Ahmed, Md Ali Hossain, Md Palash Uddin

**Affiliations:** 1 Department of Computer Science and Engineering, Hajee Mohammad Danesh Science and Technology University, Dinajpur 5200, Bangladesh; 2 Department of Computer Science and Engineering, Rajshahi University of Engineering & Technology, Rajshahi 6204, Bangladesh; 3 School of Information Technology, Deakin University, Geelong, VIC 3220, Australia

**Keywords:** hyperspectral image classification, remote sensing, feature extraction, feature selection, feature reduction, band grouping, mutual information

## Abstract

A hyperspectral image (HSI), which contains a number of contiguous and narrow spectral wavelength bands, is a valuable source of data for ground cover examinations. Classification using the entire original HSI suffers from the “curse of dimensionality” problem because (i) the image bands are highly correlated both spectrally and spatially, (ii) not every band can carry equal information, (iii) there is a lack of enough training samples for some classes, and (iv) the overall computational cost is high. Therefore, effective feature (band) reduction is necessary through feature extraction (FE) and/or feature selection (FS) for improving the classification in a cost-effective manner. Principal component analysis (PCA) is a frequently adopted unsupervised FE method in HSI classification. Nevertheless, its performance worsens when the dataset is noisy, and the computational cost becomes high. Consequently, this study first proposed an efficient FE approach using a normalized mutual information (NMI)-based band grouping strategy, where the classical PCA was applied to each band subgroup for intrinsic FE. Finally, the subspace of the most effective features was generated by the NMI-based minimum redundancy and maximum relevance (mRMR) FS criteria. The subspace of features was then classified using the kernel support vector machine. Two real HSIs collected by the AVIRIS and HYDICE sensors were used in an experiment. The experimental results demonstrated that the proposed feature reduction approach significantly improved the classification performance. It achieved the highest overall classification accuracy of 94.93% for the AVIRIS dataset and 99.026% for the HYDICE dataset. Moreover, the proposed approach reduced the computational cost compared with the studied methods.

## 1. Introduction

A hyperspectral image (HSI), which is acquired at a contiguous spectral wavelength of the electromagnetic spectrum (EM), is a rich data source for a wide range of real-world remote sensing applications, including agriculture, geology, mining, military surveillance, and others [1,2]. Moreover, an HSI is set up as a hypercube and often has hundreds of contiguous, narrow bands in the spectral image [3,4]. Due to the fact that each of these image bands contains varying intensities for the ground cover, they are each referred to as individual features [5,6,7].

There are two dimensions of spatial information and one dimension of spectral information in an HSI, which comprise the three dimensions of spectral-spatial information in the HSI (see Figure S1 in the Supplementary Files) [5,6]. Each spectral image is referred to as a feature for classification in this context, since it contains the distinct responses of the ground surface [7]. Four essential obstacles to a successful classification task are present in a high-dimensional HSI (i.e., an HSI with hundreds of image bands or features). First, because the hyperspectral sensor collects the images in continuous and contiguous spectral ranges, the neighboring image bands are highly correlated and certain image bands carry less discriminating information [5,8]. Secondly, the spectral bands are not equally important, as the bands are captured in different wavelengths of the EM spectrum [9]. Thirdly, there is a significant lack of training samples for some classes [10], which, in turn, creates the Hughes phenomenon or curse of dimensionality problem [11]. The Hughes phenomenon describes the fact that classification accuracy initially rises steadily as the number of spectral bands or dimensions rises, but falls sharply after the number of bands reaches a certain level. Finally, the computational cost of using the entire original HSI is highly expensive [7]. 

Effective feature (band) reduction is necessary to lower high-dimensional HSIs and create a suitable subspace of the features in order to improve the classification results [12,13,14,15,16] in order to address the aforementioned issues. For the accurate classification of HSIs, feature reduction (FR) techniques using feature extraction (FE) and/or feature selection (FS) might be used. FE maps the original HSI into a new space with a dimensionality of *K* from the original space with a dimensionality of P, where K ≪ P, using nonlinear or linear conversion [3]. Unsupervised and supervised procedures are the two methods of reducing dimensionality that are used most frequently. While unsupervised procedures do not make any assumptions about the existing knowledge, supervised methods are intended to preserve previously known information (ground truth). The most widely used unsupervised linear FE approach is principal component analysis (PCA) [17,18,19]. It is based on the idea that adjacent bands are highly correlated, and uses global statistics to eliminate the connections between bands [20,21]. It is often claimed that PCA is better for data compression purposes but is not suitable for extracting the most informative feature in the classification task [22,23,24,25,26,27,28,29]. The reasons for this are: (i) PCA may not catch the detailed local statistics, as it determines the overall characteristics of the entire HSI; (ii) the top principal components (PCs) or transformed features may not always contain the informative structure of the entire HSI (i.e., the tasks are biased in PCs with high variance); and (iii) PCA requires a high computational cost for high-volume hyperspectral data, as it considers the global statistics [30,31,32].

To address the pitfalls of the classical PCA, correlation-based segmented PCA (SPCA) was presented in [22], which applies conventional PCA to the bands’ subgroups. The entire dataset is divided into multiple segments using the image’s band-to-band correlation matrix. For a subgroup’s dataset, the contiguous strongly correlated bands are often assigned. However, this correlation-based segmentation strategy can only sufficiently reflect the linear relationships of the bands for making the subgroups. As such, correlation-based segmentation might not be feasible for performing classical PCA on large-volume HSIs with a huge number of bands for effective FE. Comparatively, mutual information (MI) is a dependence metric that has a built-in ability to manage the HSI in both linear and nonlinear connections [9,33]. With this motivation, we proposed a band grouping method of partitioning the spectral bands using a band-to-band normalized MI (NMI) matrix for effective FE, which is called band grouping-based PCA (BgPCA). The suggested FE method, BgPCA, first uses the NMI measure to divide the original bands into multiple groups and then applies conventional PCA separately to each subgroup of the original image bands at a minimum computing cost. 

As segmented PCA is applied to the complete dataset, there is a need to apply feature selection to select the optimal number of features. For FS, the subspace of effective features extracted by our BgPCA transformation for classification is selected using the NMI values of the transformed features to a specific range, thus meeting the minimum redundancy and maximum relevance (mRMR) criteria. Accordingly, the complete FR approach is known as BgPCA-NMI, which significantly enhances the classification performance and minimizes the computational costs as well. Although the proposed method shows outstanding performance in terms of different performance measure metrics, it has some limitations. A user-defined threshold is used to effectively partition the complete HSI. It can be optimized adaptively, and our future goal is to use a network model that automatically selects the threshold value from the dataset. On the other hand, the proposed method only addresses the spectral features. However, data redundancy exists in the spatial domain of the HSI. As such, in the future, a deep learning-based approach could be used to extract the spectral-spatial information [34,35] alongside our proposed FR technique for further improving the classification outcome. To this end, the main contributions of this study are listed below.

- We propose an MI-driven efficient FR approach for the effective classification of HSI.
- We introduce an NMI-based band grouping strategy for intrinsic FE by applying classical PCA transformation to each group of bands independently for effective FE from HSI.
- We propose an NMI-based mRMR FS method using the extracted features through our proposed transformation.
- We performed extensive experiments on two widely used benchmark HSI datasets captured by the AVIRIS and HYDICE sensors to validate the superiority of our proposed FR approach.

We have organized the rest of the article as follows. In Section 2, we first describe the insights of the proposed NMI-based band grouping strategy for applying classical PCA in a segmented manner. Next, the proposed FE called BgPCA is elaborately presented. After that, we discuss the NMI-based mRMR FS criteria on top of our BgPCA transformation. Lastly, we present the complete FR method called BgPCA-NMI at the end of Section 2. In Section 3, we intricately analyze the experiments conducted on two real HSI datasets using the proposed BgPCA-NMI FR approach and the state-of-the-art methods. Finally, Section 4 summarizes the outcomes and concludes the article. 

## 2. Methodology

**Figure 1:**
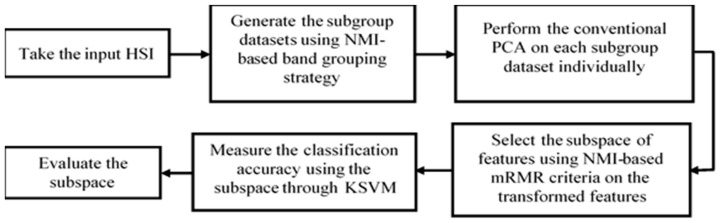
The workflow of the proposed BgPCA-NMI.

The proposed FR approach, called BgPCA-NMI, encompasses three main steps: (i) band grouping based on the band-to-band NMI matrix of the entire original HSI; (ii) FE through implementation of classical PCA on each subgroup dataset independently; and (iii) FS through measurement of the NMI-based mRMR criteria of the transformed features of the HSI. Figure 1 illustrates the working steps of our BgPCA-NMI.

### 2.1. Proposed Band Grouping Strategy Based on NMI

The MI is a popular information-theoretic metric used to measure the general dependency of two random variables, say two image bands A and B. The MI, denoted I(A,B), is defined as

(1) $$I\left(A,B\right)=\sum_{b\in B}\sum_{a\in A}p\left(a,b\right)\log\frac{p\left(a,b\right)}{p\left(a\right)\;p\left(b\right)}$$

where p(.) denotes the marginal probability and p(., .) is the distribution of the joint probability. In the context of HSI, the MI assesses the information that is shared among the spectral image bands and intuitively determines the interdependence among the image bands. A higher MI indicates more dependency between them and vice versa. Note that although correlation has been successfully applied as a similarity measure tool in many studies [22,36,37], it suffers when nonlinearity exists in the image bands. Unlike the correlation metric, the MI finds the dependency of the image bands both in linear and nonlinear ways. However, the MI value is not bound to a precise range, which creates difficulty when measuring the actual relationships of the relevant image bands of the HSI. Therefore, the MI value can be mapped to a specific range for normalizing the measure as follows [38]

(2) $$\;\hat{I}\left(A,B\right)=\frac{I\left(A,B\right)}{\sqrt{H\left(A\right)H\left(B\right)}}$$

**Figure 2:**
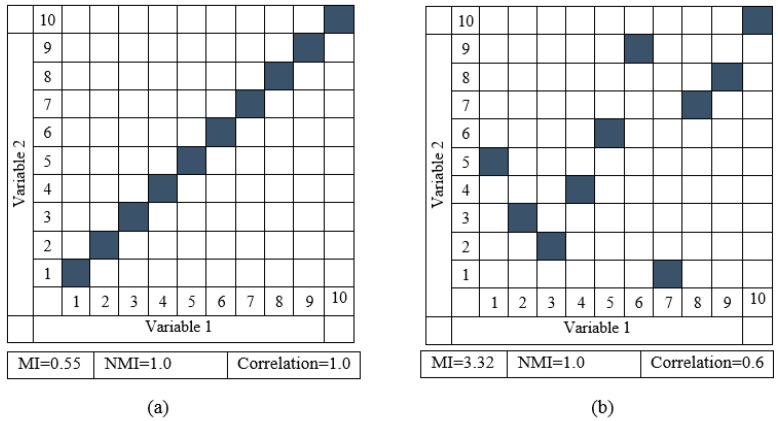
Scatterplots illustrating two variables in (**a**) a perfect linear connection and (**b**) a nonlinear point-to-point relationship.

In Equation (2), $\;\hat{I}\left(A,B\right)$ is the NMI between the image bands A and B, and H(.) denotes the marginal entropy. Figure 2 represents how the NMI measure is more influential compared with the correlation measure and traditional MI. Figure 2a depicts the scatterplots of two random variables showing a perfect linear relationship, and Figure 2b illustrates the scatterplots expressing a point-to-point but nonlinear relationship between the variables. It can be observed that the correlation is strong when the variables are fully linear and low when the variables are nonlinear. Contrariwise, the NMI is high (i.e., 1.0) for both cases, which reemphasizes the superiority of the NMI-based similarity measure.

**Figure 3:**
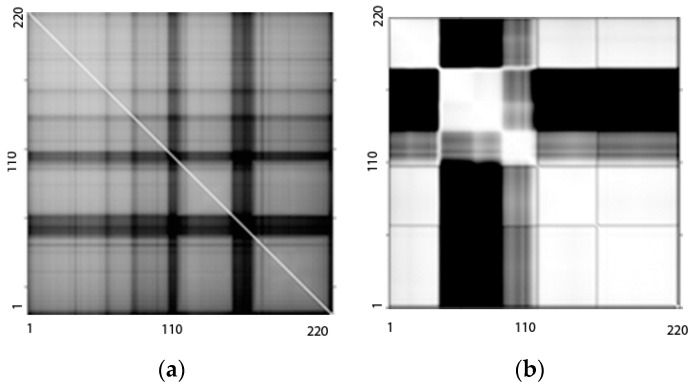
Spectral band-to-band measurement used for segmentation of the Indian Pines HSI: (**a**) NMI matrix in the image and (**b**) the correlation matrix in image form.

We determined the band-to-band NMI matrix for the dataset by computing the NMI between every pair of HSI bands in order to segment the complete HSI. As an illustration, Figure 3a shows the baseline correlation matrix image notation and Figure 3b shows the NMI matrix in image notation of the AVIRIS Indian Pines dataset. The NMI values of the HSI bands that are close to one another are greater than those of the bands that are farther apart. The HSI bands from the original data cube were therefore divided and grouped using the NMI matrix image. The details of the subgroups using this proposed NMI-based segmentation strategy and the baseline correlation-based segmentation strategy of SPCA for the AVIRIS and HYDICE datasets are shown in Table 1 and Table 2, respectively.

### 2.2. PCA

In order to extract meaningful information, PCA was used to calculate the relationships between the spectral image bands in HSI. This depended on the fact that the HSI’s neighboring bands were strongly linked and frequently communicate information about the ground entities that were similar to one another [28,29]. Let the spectral vector, denoted as $X_{n}$, in X be defined as $X_{n}={\left[X_{n1}X_{n2}\ldots X_{nP}\right]}^{T}$, where $n\in \left[1\;S_{\mathrm{all}}\right]$. The mean adjusted spectral vector, $I_{n}$, can now be obtained as follows

(3) $$I_{n}=X_{n}-M$$

where the mean image vector, $M=\frac{1}{S_{\mathrm{all}}}\overset{S_{\mathrm{all}}}{\underset{n=1}{\sum }}X_{n}$. The zero-mean image, denoted I, is thus obtained as $I=\left[I_{1}I_{2}\ldots I_{n}\right]$. After that, the covariance matrix, C is computed as follows

(4) $$C=\frac{1}{S_{\mathrm{all}}}I\;I^{T}$$

Eigenvalues $E=\left[E_{1}E_{2}\ldots E_{P}\right]$ and eigenvectors $V=\left[V_{1}V_{2}\ldots V_{P}\right]$ are obtained by disintegrating the covariance matrix C as $C=VEV^{T}$. The orthonormal matrix, Z**,** is collected by picking K eigenvectors after reorganizing the eigenvectors with the peak eigenvalues, where K<P and often K≪P. Finally, the transformed or projected data matrix, Y, is calculated as

(5) $$Y=Z^{T}I$$

### 2.3. Proposed BgPCA

When the multispectral dataset has a small number of distinct bands, it has been observed that conventional PCA is practical and delivers satisfactory results when extracting appropriate features [19]. Applying traditional PCA to the complete dataset for hyperspectral images may provide biased outcomes in addition to an exponential rise in processing time and computing expense [22]. The highly connected image bands of the HSI, however, appear in blocks and were shown to be highly associated with respect to the bands that are closer together. Additionally, PCA retrieves the HSI data while taking the overall HSI features into account and fails to extract the local information. In order to eliminate the less correlated bands between the highly correlated blocks, SPCA changes the use of traditional PCA. 

Because it cannot handle the nonlinear interactions between the bands during the segmentation phase, the correlation-based segmentation of HSI utilized in SPCA may still be impractical. As such, we proposed an NMI-based band grouping mechanism for more efficient band segmentation to handle both linearity and nonlinearity in the image bands, which was discussed in Section 2.1. The improvement allowed by the proposed BgPCA over conventional PCA is that it extracts the local characteristics of data in an efficient way rather than considering the global statistics of the HSI. Moreover, the computational cost of conventional PCA can be significantly reduced with BgPCA and, consequently, the total computational cost of HSI classification is decreased.

**Figure 4:**
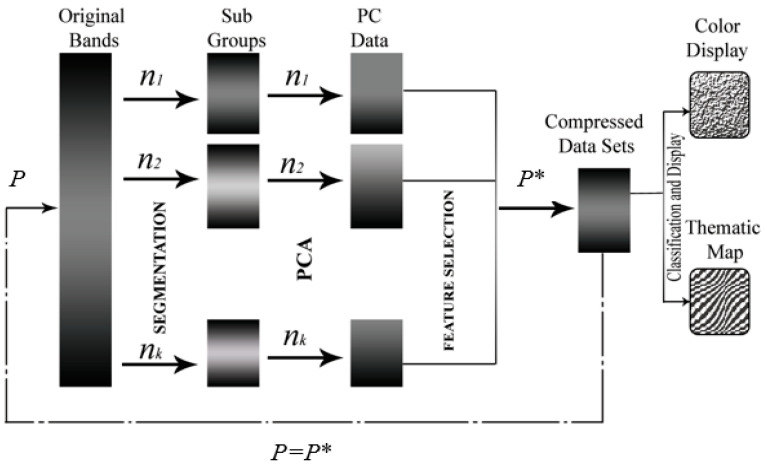
Working stages of BgPCA, P* denotes the number of transformed features.

In the implementation of BgPCA, the complete HSI dataset is separated into k subgroup datasets based on the NMI-based segmentation scheme. Next, the covariance matrix is computed for each subgroup’s dataset. Afterward, each estimated covariance matrix of the distinct subgroups is subjected to the eigen-decomposition procedure independently. Consequently, the final projection matrix of the entire dataset is found by merging the individual projection matrices consecutively. We pictorially illustrate the working principle of the proposed BgPCA in Figure 4, and the pseudocode is given in Algorithm 1.
**Algorithm 1.** BgPCA
**Start** {**X**: the 2D dataset of the HSI}**Calculate** the band-to-band NMI matrix to produce the subgroups of **X**Based on the NMI matrix, **divide X** into *k* subgroups**For each** subgroup, **do****Compute** the projection matrix via PCA**Combine** all individual projection matrices successively to build the complete projection matrix

### 2.4. Proposed BgPCA-NMI

For selecting the effective subspace of the extracted features, we first measured the MI between each new extracted feature, $Y_{i}$, from our BgPCA transformation and the ground truth image, T. Accordingly, the most informative feature could be calculated using Equation (6) and assigned to the feature subspace, S.

(6) $$V=\max_{i\in P}\;I(Y_{i},T).$$

The K features selected by using Equation (6) can have potential redundancy, which would affect the classification performance. As such, the redundancy between the selected features needed to be minimized for efficient classification. To address this, we applied the mRMR criteria for selecting K effective features as follows

(7) $$G\left(Y_{i},K\right)=I\left(Y_{i},T\right)-\frac{1}{K}\underset{s_{j}\in S}{\sum }I\left(Y_{i},s_{j}\right),Y_{i}⊄S$$

Since it is impacted by the entropy of two variables and is not constrained to a certain range, the MI value, $I\left(Y_{i},T\right)$ from the equation above is challenging to utilize directly. To measure how good an MI value is, we normalized it to the range [0, 1] using Equation (8), while the proposed informative FS approach is defined in Equation (9).

(8) $$\;\hat{I}\left(Y_{i},T\right)=\frac{I\left(Y_{i},T\right)}{\sqrt{H\left(Y_{i}\right)H\left(T\right)}}$$

(9) $$\;\hat{G}\left(Y_{i},K\right)=I\left(Y_{i},T\right)-\frac{1}{K}\underset{s_{j}\in S}{\sum }\;\hat{I}\left(Y_{i},s_{j}\right),Y_{i}⊄S$$

Equation (9), when applied, might result in a difference between the selected characteristics and those that have previously been chosen, which is undesirable. Therefore, $\;\hat{G}\left(Y_{i},K\right)$ was considered to be positive, i.e., $\;\hat{G}\left(Y_{i},K\right)>0$, in our analysis. The complete pseudocode is given in Algorithm 2.
**Algorithm 2.** BgPCA-NMI
**Start** {**Y**: the projected data matrix via BgPCA}**Instantiate** the feature subspace to null, **S**_0_ = {Φ}**Choose** the first feature, **Y***_j_* using Equation (6) via utilizing Equation (8) and execute $S_{1}=S_{0}\;\cup \;Y_{j}$**For** choosing the rest of the features, **do****Apply** Equation (9) and update **S****Take S** as the subspace of the top effective features

## 3. Experiment and Analysis of the Results 

### 3.1. Description of the Dataset 

In the experiment, the classification task used two different benchmark HSI datasets. We leveraged the dataset from an urban mall in Washington, DC, and the mixed agricultural Indian Pines dataset, which were captured by the AVIRIS and HYDICE sensors, respectively. The Indian Pines HSI has 220 spectral imaging bands in total, each with a spatial resolution of 145 × 145 pixels [39]. However, due to the impact of atmospheric phenomena, its 20 water absorption bands ([104, 108], [150, 163], and 220) have been disregarded in this analysis. For classification purposes, there w 16 classes in the ground truth map. The “grass/mown pasture” and “oats” classes were not utilized in this experiment, since there were insufficient data for them. A high-volume dataset with a spatial resolution of 1280 × 307 pixels, the urban Washington DC Mall dataset, comprises 191 spectral image bands [40]. The ground truth map has seven classes available. The “paths” class was not utilized for classification in the experimental analysis, as there were insufficient samples. We display some sample band images and the ground truth image of the Indian Pines HSI and the false color image of the Washington DC Mall HSI with its ground truth information in Figure S2 in the Supplementary Files, while Table 3 illustrates the key properties of these datasets. 

### 3.2. Results of FE and FS 

The SPCA transformation partitioned the original HSI into several subgroups using the correlation matrix; however, the suggested FE method BgPCA divided the image bands using the NMI matrix. The results of BgPCA segmentation shown in Table 1 are based on the findings obtained by accounting for NMI values greater than the threshold of 0.3 and looking for edges in the image of the NMI matrix (Figure 3a) together with the diagonal direction. The total number of subgroup datasets that will be created is indicated by the user-defined threshold when the threshold is low, the number of subgroups increases, and uncorrelated bands may be clustered together. We chose the threshold value so that the image bands with high correlations were grouped together. To carry this out, we looked at the dataset and the NMI matrix image, and used the trial-and-error method to choose the threshold value. The entire AVIRIS dataset was split into three subgroups in Table 1, which each contained 102, 41, and 57 bands. However, the correlation-based SPCA also partitioned the entire dataset into three subgroups consisting of 35, 68, and 97 bands. Similarly, we partitioned the whole HYDICE dataset into four subgroup datasets comprising 58, 50, 51, and 33 bands for BgPCA, as illustrated in Table 2. Nevertheless, SPCA divided the entire HYDICE dataset into three highly correlated subgroups consisting of 56, 46, and 89 bands. PCA was applied separately to each individual subgroup for both BgPCA and SPCA with both datasets. For both BgPCA and SPCA, the informative features were selected using the NMI-based mRMR criteria for effective subspace detection. Table S1 in the Supplementary Files presents the acronyms associated with the proposed method and the different methods studied, while the order of ranked features that were used for classification is listed in Tables S2 and S3 in the Supplementary Files for the Indian Pines and Washington DC datasets, respectively. In Tables S2 and S3, the segmentation number is utilized first, followed by the PC number of this segment (Segment:PC) for the segmented PCA (SPCA) technique. The proposed BgPCA approach initially gives the group number, followed by the number of PCs in this group (Group:PC). 

### 3.3. Performance Evaluation Metrics

The overall accuracy (OA), average accuracy (AA), Kappa coefficient, and F1 score are widely used quality indices applied in this study to evaluate how well the proposed technique performed. The percentage of all correctly identified pixels is known as the OA, and it can be calculated as follows:

(10) $$\mathrm{OA}=\overset{C}{\underset{i=1}{\sum }}\frac{A_{ii}}{B}$$

In Equation (11), *C* stands for the number of classes and *A* for the confusion matrix, which is determined by contrasting the classification map with the ground truth image. The number of samples belonging to Class *i* and labeled as Class *i* (i.e., values found along the diagonal of the confusion matrix) is represented by *A_ii_*, whereas the total number of test samples is represented by *B*. 

AA stands for the average accuracy, which is the average proportion of correctly classified pixels for each class, which is determined as follows

(11) $$\mathrm{AA}=\frac{\sum_{i=1}^{C}\left(A_{ii}/\sum_{i=1}^{C}A_{i+}\right)}{C}$$

where *A_ii_* stands for total number of samples belonging to Class *i* and classified as Class *i* (i.e., values found along the diagonal of the confusion matrix), and *A_i+_* represents the total number of samples as classified as Class *i.*

The Kappa coefficient computes the proportion of classified pixels adjusted for the number of agreements predicted only by chance. The Kappa statistic indicates how much better the categorization performs than the likelihood of randomly assigning pixels to their correct categories and can be calculated using the notation used in Equations (10) and (11) as

(12) $$\mathrm{Kappa}=\frac{\left(B\sum_{i=1}^{C}A_{ii}-\sum_{i=1}^{C}(A_{i+})\left(A_{+i}\right)\right)}{\left(B^{2}-\sum_{i=1}^{C}\sum_{i=1}^{C}(A_{i+})\left(A_{+i}\right)\right)}$$

where *A_+i_* represents the total number of actual samples in Class *i.* The F1 score can now be calculated as follows

(13) $$F1\;\mathrm{score}=\frac{2\times \mathrm{Precision}\times \mathrm{Recall}}{\mathrm{Precision}+\mathrm{Recall}}$$

where the precision and recall can be calculated as follows:

(14) $$\mathrm{Precision}=\frac{\mathrm{TP}}{\mathrm{TP}+\mathrm{FP}}\;\mathrm{and}\;\mathrm{Recall}=\frac{\mathrm{TP}}{\mathrm{TP}+\mathrm{FN}}$$

Here, TP, FP, and FN denote the number of true positive, false positive, and false negative classifications of the testing samples of multiple classes, respectively. 

### 3.4. Classification Results and Evaluation

The performance of the proposed FR method, BgPCA-NMI, was assessed in terms of the following classical performance measure metrics: OA, AA, Kappa, precision, recall, and F1 score. We also considered a scatterplot-based feature space analysis scheme and the computation cost for better expressing the robustness of our BgPCA-NMI FR method. After FE, the first few ranked features selected by the FS approach, as illustrated in Tables S2 and S3 in the Supplementary Files, were used to calculate the abovementioned performance measure metrics using the kernel support vector machine (KSVM) and the radial basis kernel function (RBF) [41] to tackle any nonlinearity in the final feature set. For the task of efficient training, 10-fold cross-validation with a grid search strategy was used for selecting the best cost parameter (C_cost) and kernel width (γ) associated with the RBF-KSVM. The proposed FR approach was compared with conventional PCA and correlation-based SPCA with variance, and with NMI-based mRMR FS and BgPCA with variance-based feature ranking. The kernel parameters C_cost = 8 and γ = 1.33 for the AVIRIS dataset and C_cost = 6 and γ = 1.23 for the HYDICE dataset were tuned by using 15 features of the AVIRIS dataset and 8 features of the HYDICE dataset. Table 4 shows all the parameter tuning results for the KSVM classifier of the proposed and other algorithms studied for the two HSI datasets. In total, 2127 pixels from 14 different classes from the AVIRIS dataset were used for the classification, where around 50% were used for training and 50% were used for testing, as shown in Table S4 in the Supplementary Files. On the other hand, 4464 pixels in total from six classes from the HYDICE dataset were used for classification, where 30% were used for training and 70% were used for testing, as illustrated in Table S5 in the Supplementary Files.

**Figure 5:**
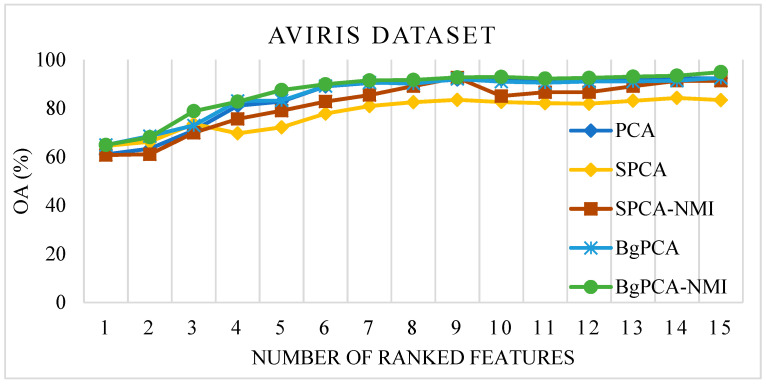
Overall classification accuracy on the AVIRIS dataset.

**Figure 6:**
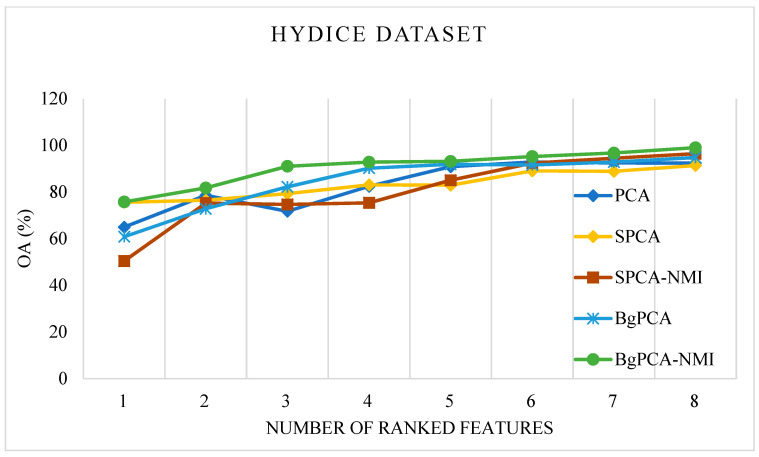
Overall classification accuracy on the HYDICE dataset.

For the AVIRIS Indian Pines dataset, the OAs produced by the studied methods (PCA, SPCA, and SPCA-NMI) and the proposed approach (BgPCA-NMI along with plain BgPCA) are depicted in Figure 5. In this case, a line graph was utilized to assess the relevance of the ranking attributes at each stage. To begin, just the top-ranked feature was utilized to calculate the classification accuracy. The top two features were then used and calculated. Following that, the classification accuracy was computed using the first three characteristics. In this manner, the total classification accuracy was determined and shown in the line graph using the selected features. The traditional PCA and correlation-based SPCA achieved 92.45% and 83.40% OA, respectively. On the other hand, the proposed FR method, BgPCA-NMI, had the highest OA of 94.93% using the same number of features, which clearly indicates the advantage of using the proposed approach. For the HYDICE Washington DC Mall dataset, the OAs for the different methods are illustrated in Figure 6. The OAs produced by PCA and SPCA were 92.8% and 94.81%, respectively, whereas the proposed FR method, BgPCA-NMI, had the highest OA of 99.026% using the same number of features. This also led to the superiority of using the proposed feature space identification over the existing methods.

Table 5 and Table 6 show all the classification performance metrics (AA, OA, Kappa, and F1 score) for the proposed FR method and each of the other studied methods. The proposed method demonstrated an improvement in terms of all these metrics as compared with the studied methods. The robustness of the proposed method, BgPCA-nMI, for multiclass classification was also evaluated using the error matrices, as shown in Tables S6 and S7 for the AVIRIS and HYDICE datasets, respectively, in the Supplementary Files. From both error matrices, it can be seen that almost all classes were correctly predicted, except for very few of them.

**Figure 7:**
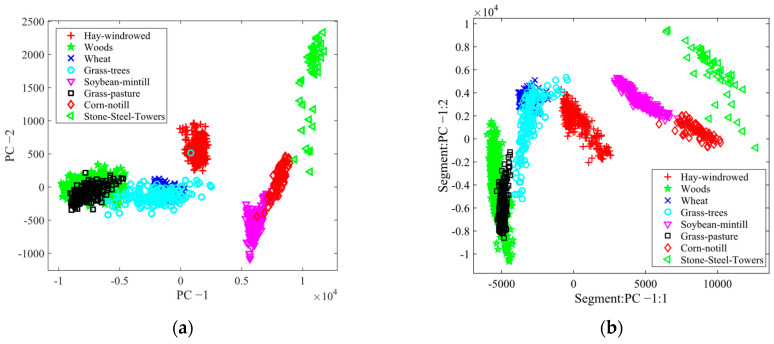

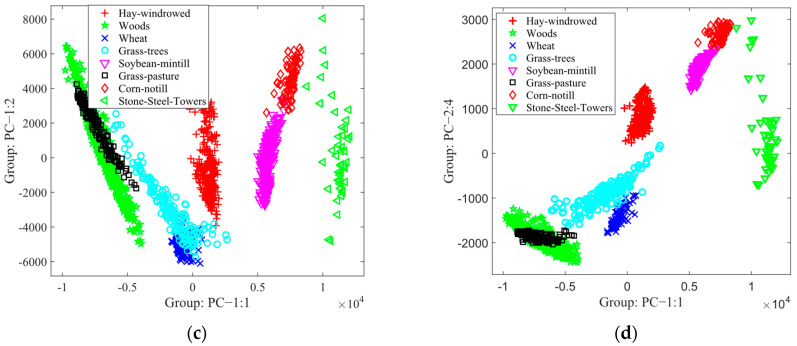
Scatterplots for different methods for the Indian Pines dataset: (**a**) PCA, (**b**) SPCA, (**c**) BgPCA, and (**d**) BgPCA-NMI.

**Figure 8:**
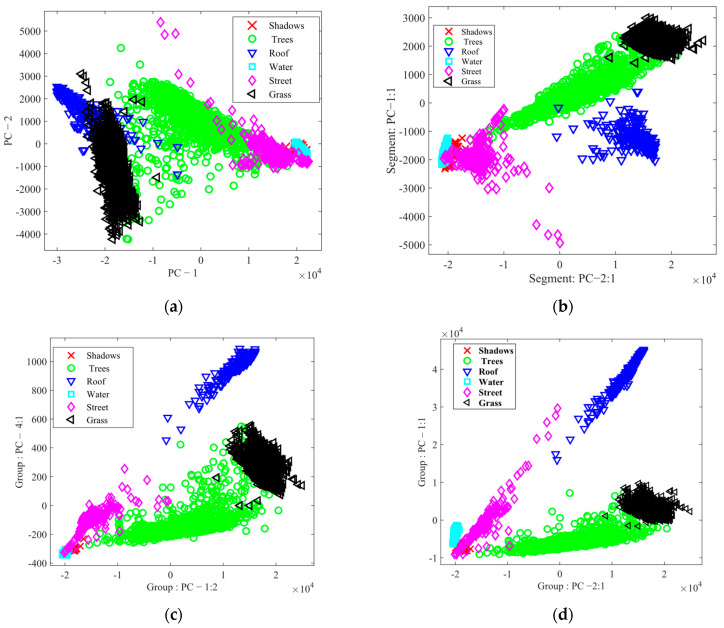
Scatterplots for different methods for the Washington DC Mall dataset: (**a**) PCA, (**b**) SPCA, (**c**) BgPCA, and (**d**) BgPCA-NMI.

By utilizing the feature space analysis framework, we evaluated the robustness of the suggested method, BgPCA-NMI. Figure 7 depicts the 2D feature space for the AVIRIS Indian Pines HSI using the first two ranked features for each method to show the effects of feature selection and feature extraction. For simplicity, eight classes were plotted in the feature space. Visually, it is clear that the proposed FR method separated the classes better than the tested methods. Similarly, Figure 8 shows the 2D feature space for the Washington DC Mall dataset using the first two ranked features. The outcome further proved that the suggested FR strategy separated the classes more effectively than the approaches under investigation. It was therefore observed that the scatterplots represented how the classes were separated from one another. For the proposed method, we can see that the classes are more separated compared with the studied methods. If the classes are separated well, the method will classify the samples more accurately. Note that we could not find any other additional statistics to calculate these classes regarding the transformed spaces from the literature on hyperspectral imagery.

The effectiveness of the proposed approach, BgPCA-NMI, was finally assessed using the total computational time in different stages, as given in Table 7, for both datasets. The proposed method was tested on a personal computer equipped with an Intel Core i5 3.2 GHz CPU and 8 GB of RAM, running on the Microsoft Windows 10 operating system. It was evident that the suggested approach, BgPCA-NMI, took less time overall to compute than conventional PCA, indicating an increase in computational efficiency.

## 4. Conclusions and Future Work

Because an HSI is a high-dimensional data cube, effective FE is necessary to provide outstanding classification performance while decreasing the computing costs. In this study, we used the NMI measure because of its appropriate treatment of nonlinearity in partitioning the original HSI bands efficiently instead of using the correlation for the segmentation, as in the case of SPCA. For successful FE, PCA was performed on each subgroup of bands after the band-to-band NMI matrix of the HSI had been utilized to divide all the spectral bands into a number of groups. As a result, the proposed FE approach extracted useful features while taking the HSI dataset’s local characteristics into account, and the computational cost of extracting the features decreased greatly. After that, the NMI between each transformed feature and the ground truth was used for selecting the subspace of informative features using the mRMR scheme. In comparison with traditional PCA and correlation-based SPCA, BgPCA-NMI increased the classification accuracy, as shown by the classification performance and analysis of the results on two actual HSI datasets, Indian Pines and Washington DC Mall. Ultimately, the proposed method, BgPCA-NMI, effectively reduced the computational cost. 

Effective partitioning of the whole HSI was achieved by using a user-defined threshold. We want to utilize a network model that automatically chooses the threshold value from the dataset in the future. It may be optimized adaptively. On the other hand, the proposed method just takes the spectral characteristics into account. However, there is data redundancy in the HSI’s spatial domain. As a result, in the future, our suggested FR technique, as well as a deep learning-based strategy, will be used to extract the spectral and spatial information to further improve the classification results. Finally, as well as our feature space analysis, other distance metrics or statistics, such as the Bhattacharyya distance, class compactness, etc., within the PC space and BgPCA space could be used in the future to quantify the separation better.

## Figures and Tables

**Table 1:** Band grouping information of the Indian Pines (AVIRIS) HSI.

	SPCA (Baseline Approach)			BgPCA (Proposed Approach)		
Group	Range of Bands	# of Bands	Average Correlation	Range of Bands	# of Bands	Average NMI
1	1–35	35	0.8770	1–102	102	0.3400
2	36–103	68	0.7171	103–143	41	0.7171
3	104–200	97	0.6950	144–200	67	0.6950

**Table 2:** Band grouping information of the Washington DC (HYDICE) HSI.

	SPCA (Baseline Approach)			BgPCA (Proposed Approach)		
Group	Range of Bands	# of Bands	Average Correlation	Range of Bands	# of Bands	Average NMI
1	1–56	56	0.9443	1–58	58	0.4500
2	57–102	46	0.8842	59–108	50	0.5460
3	103–191	89	0.9813	109–159	51	0.5460
4	-	-	-	159–191	33	0.5600

**Table 3:** Summary of the datasets.

Name of the Dataset	Capturing Sensor	*P*	Wavelength Range (nm)	*H*	*W*	Ground Classes	Ground Sampling Distance (m)
Indian Pines	AVIRIS	220	400–2500	145	145	16	20
Washington DC Mall	HYDICE	191	400–2400	1280	307	7	3

**Table 4:** Parameter tuning using 10-fold cross-validation.

	Method Name	Best C	Best γ	Training Accuracy
AVIRIS	PCA	10	3	98.55
	SPCA	3.5	2.8	94.65
	SPCA-NMI	5	2	98.50
	BgPCA	1.8	3.7	96.85
	BgPCA-NMI	7	1.2	98.88
HYDICE	PCA	10	3	97.55
	SPCA	4	2.1	95.83
	SPCA-NMI	2.5	3.9	97.68
	BgPCA	3	1.5	98.15
	BgPCA-NMI	7	1.2	98.95

**Table 5:** Detailed classification outcomes for the Indian Pines dataset.

Class	PCA	SPCA	SPCA-NMI	BgPCA	BgPCA-NMI
Hay—windrowed	97.66	90.60	90.60	94.41	96.43
Soybean—no till	80.41	76.67	94.81	84.54	84.69
Woods	94.96	92.27	91.27	96.19	96.71
Wheat	100.00	98.41	100	94.03	100.00
Grass—trees	100.00	100.00	100	100	100.00
Soybean– min. till	89.67	70.21	92.64	94.34	94.94
Grass—pasture	94.74	60.00	90.00	77.42	85.71
Corn –no till	96.67	95.24	95.12	100	100.00
Corn	88.10	92.68	76.47	88.00	97.78
Corn—min. till	100.00	100.00	68.75	87.50	100.00
Stone, steel, towers	100.00	100.00	77.27	100	100.00
Alfalfa	54.55	100.00	100	100	100.00
Soybean—clean	100.00	69.23	83.33	100	100.00
Buildings, grass, trees, roads	80.00	75.00	83.33	88.89	81.82
AA	91.20	87.17	88.83	93.24	95.58
OA	92.45	83.40	91.35	92.54	94.93
Kappa	91.24	80.77	90.00	91.41	94.16
Precision	80.23	70.45	82.22	82.23	91.87
Recall	91.20	87.17	88.83	93.24	95.58
F1 score	85.36	77.92	85.40	87.95	93.69

**Table 6:** Detailed classification outcomes for the Washington DC Mall dataset.

Class	PCA	SPCA	SPCA-NMI	BgPCA	BgPCA-NMI
Shadow	34.04	57.14	59.26	72.73	88.89
Tree	99.27	99.88	99	99.79	99.81
Roof	100	99.05	99.08	100	100
Water	100	100	100	100	100
Street	93.63	81.51	89.41	83.86	97.62
Grass	69.12	86.46	87.42	95.82	98.26
AA	95.05	87.34	89.03	92.03	97.43
OA	92.80	92.07	93.57	95.97	99.03
Kappa	90.22	89.32	91.30	94.54	98.67
Precision	93.62	95.49	96.79	97.77	99.51
Recall	95.05	87.34	89.02	92.03	97.43
F1 score	94.33	91.23	92.75	94.81	98.46

**Table 7:** Computational time in seconds (s) of the proposed and studied methods.

Stage	AVIRIS		HYDICE	
	PCA	BgPCA-NMI	PCA	BgPCA-NMI
FE	0.098 s	0.017 s	0.120 s	0.067 s
FS	1.200 s	0.980 s	1.100 s	0.670 s
Total cost	1.298 s	0.997 s	1.220 s	0.737 s

## Data Availability

The AVIRIS Indian Pines data [39] are available at https://purr.purdue.edu/publications/1947/1 (accessed on 3 March 2022), while the HYDICE Washington DC Mall data [40] are available at https://engineering.purdue.edu/~biehl/MultiSpec/hyperspectral.html (accessed on 3 March 2022).

## Supplementary Materials

The following supporting information can be downloaded at: https://www.mdpi.com/article/10.3390/s23020657/s1, Figure S1: Structure of an HSI; Table S1: Acronyms of different studied and proposed methods; Figure S2: HSI datasets; Table S2: The rank of selected features for the classification of Indian Pines dataset; Table S3: The rank of selected features for the classification of DC Mall dataset; Table S4: Training and testing samples for AVIRIS dataset; Table S5: Training and testing samples for HYDICE dataset; Table S6: Error matrix of BgPCA-NMI for Indian Pines dataset; Table S7: Error matrix of BgPCA-NMI for DC Mall dataset.
