# Supplementary material for: Mutual Information-Driven Feature Reduction for Hyperspectral Image Classification
Source: Sensors (Basel). 2023 Jan 6;23(2):657. doi: 10.3390/s23020657 (PMC9864404; doi:10.3390/s23020657)
Supplement: Supplementary file 1 [file sensors-23-00657-s001.zip › sensors-2068904-supplementary.pdf]

sensors

Supplementary Material

Mutual Information-Driven Feature Reduction for Hyperspectral Image Classification

Md Rashedul Islam [1], Boshir Ahmed [2], Md Ali Hossain [2] and Md Palash Uddin [1,3,*]

[1] Department of Computer Science and Engineering, Hajee Mohammad Danesh Science and Technology University, Dinajpur 5200, Bangladesh
[2] Department of Computer Science and Engineering, Rajshahi University of Engineering & Technology, Rajshahi 6204, Bangladesh
[3] School of Information Technology, Deakin University, Geelong, VIC 3220, Australia
* Correspondence: m.uddin@deakin.edu.au or palash_cse@hstu.ac.bd

List of supporting materials

Figure S1: Structure of an HSI.
Figure S2: HSI datasets
Table S1: Acronyms of different studied and proposed methods.
Table S2: The rank of selected features for the classification of Indian Pines dataset.
Table S3: The rank of selected features for the classification of DC Mall dataset
Table S4: Training and testing samples for AVIRIS dataset.
Table S5: Training and testing samples for HYDICE dataset.
Table S6: Error matrix of BgPCA-NMI for Indian Pines dataset.
Table S7: Error matrix of BgPCA-NMI for DC Mall dataset.

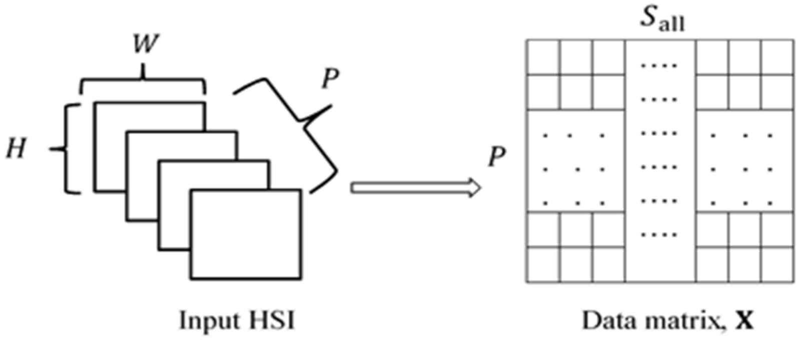

Figure S1. Transformation of an HSI (left) into a data matrix, $\mathbf{X}$ of size $P \times S_{\text{all}}$ (right), where $S_{\text{all}} = H * W$, indicating total pixels or spectral signatures in the HSI.

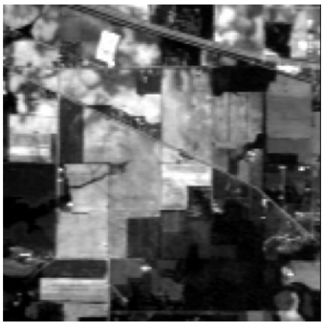

(a) Band-22 image of Indian Pines HSI

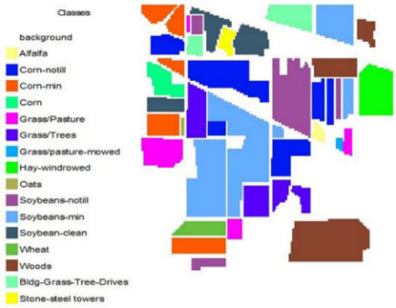

(b) Ground truth image of Indian Pines HSI

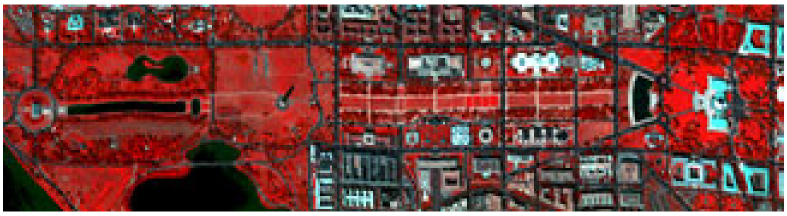

(c) False-color composite of original Washington DC Mall HSI

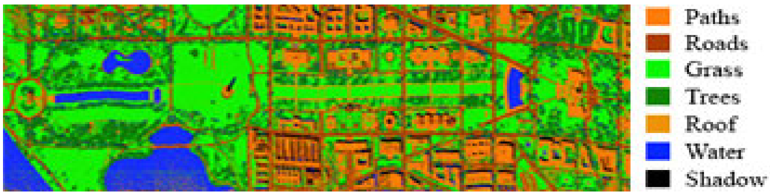

(d) Related ground truth of Washington DC Mall HSI

Figure S2. HSI datasets: (a) band-22 image of Indian Pines HSI, which is captured at 0.6 m wavelengths, (b) ground truth image of Indian Pines dataset, (c) false-color composite of original Washington DC Mall HSI, and (d) ground truth of Washington DC Mall dataset.

Table S1. Acronyms of different studied and proposed methods.

Acronym	Method
PCA	The conventional PCA
SPCA	Correlation-based band segmentation with variance-based feature ranking
SPCA-NMI	Correlation-based band segmentation with NMI-based mRMR FS
BgPCA	NMI-based band grouping with variance-based feature ranking
BgPCA-NMI	NMI-based band grouping with NMI-based mRMR FS

Table S2. The rank of selected features for the classification of Indian Pines dataset.

Method	Order of Selected Features
PCA	PC 1 to PC 15
SPCA	Segment 1: PC 1; Segment 1: PC 2; Segment 3: PC 1; Segment 2: PC 2; Segment 2: PC 3; Segment 2: PC 1; Segment 1: PC 3; Segment 1: PC 4; Segment 2: PC 3; Segment 3: PC 2; Segment 1: PC 5; Segment 1: PC 6; Segment 1: PC 7; Segment 2: PC 8; Segment 2: PC 5;

SPCA-NMI	Segment 1: PC 1; Segment 1: PC 10; Segment 1: PC 9; Segment 1: PC 3; Segment 1: PC 19; Segment 2: PC 34; Segment 1: PC 2; Segment 1: PC 5; Segment 1: PC 6; Segment 3: PC 17; Segment 1: PC 12; Segment 1: PC 7; Segment 1: PC 15; Segment 3: PC 2; Segment 2: PC 5;
BgPCA	Group 1: PC 1; Group 1: PC 2; Group 2: PC 2; Group 1: PC 3; Group 3: PC 2; Group 1: PC 4; Group 1: PC 5; Group 1: PC 6; Group 1: PC 7; Group 1: PC 8; Group 1: PC 9; Group 1: PC 10; Group 1: PC 11; Group 2: PC 3; Group 1: PC 12;
BgPCA-NMI	Group 1: PC 1; Group 2: PC 4; Group 2: PC 2; Group 2: PC 3; Group 3: PC 2; Group 1: PC 3; Group 1: PC 4; Group 3: PC 3; Group 1: PC 5; Group 1: PC 6; Group 1: PC 2; Group 3: PC 4; Group 2: PC 5; Group 1: PC 10; Group 3: PC 5;

Table S3. The rank of selected features for the classification of DC Mall dataset.

Method	Order of Selected Features
PCA	PC 1 to PC 8
SPCA	Segment 2: PC 1; Segment 1: PC 1; Segment 3: PC 1; Segment 1: PC 2; Segment 2: PC 2; Segment 2: PC 3; Segment 1: PC 3; Segment 1: PC 4;
SPCA-NMI	Segment 2: PC 1; Segment 3: PC 3; Segment 1: PC 3; Segment 1: PC 2; Segment 3: PC 1; Segment 3: PC 2; Segment 3: PC 4; Segment 1: PC 1;
BgPCA	Group 1: PC 2; Group 4: PC 1; Group 4: PC 2; Group 2: PC 5; Group 2: PC 1; Group 3: PC 3; Group 1: PC 1; Group 3: PC 2;
BgPCA-NMI	Group 2: PC 1; Group 1: PC 1; Group 1: PC 2; Group 3: PC 1; Group 2: PC 2; Group 1: PC 3; Group 1: PC 4; Group 2: PC 2;

Table S4. Training and testing samples for AVIRIS dataset.

Class	Training Samples	Testing Samples
Hay-windrowed (C1)	165	135
Soybean-notill (C2)	109	85
Woods (C3)	279	248
Wheat (C4)	42	63
Grass-trees (C5)	96	80
Soybean-mintill (C6)	130	165
Grass-pasture (C7)	108	72
Corn-notill (C8)	48	40
Corn (C9)	48	44
Corn-mintill (C10)	15	14
Stone-Steel-Towers (C11)	25	20
Alfalfa (C12)	15	15
Soybean-clean (C13)	21	10
Buildings-Grass-Trees-Drives (C14)	20	15
Total Samples	1137	1006

Table S5. Training and testing samples for HYDICE dataset.

Class	Training Samples	Testing Samples
Shadow (C1)	20	16
Tree (C2)	367	1056
Roof (C3)	117	108
Water (C4)	425	476
Street (C5)	299	369
Grass (C6)	361	850
Total samples	1589	2875

Table S6. Error matrix of BgPCA-NMI for Indian Pines dataset.

		Predicted Class													
		C1	C2	**C3**	**C4**	**C5**	**C6**	**C7**	**C8**	**C9**	**C10**	**C11**	**C12**	**C13**	**C14**
**Original Class**	C1	135	0	0	0	0	0	0	0	0	0	0	5	0	0
	C2	0	83	0	0	0	15	0	0	0	0	0	0	0	0
	C3	0	0	235	0	2	0	0	0	0	0	0	0	0	6
	C4	0	0	0	63	0	0	0	0	0	0	0	0	0	0
	C5	0	0	0	0	77	0	0	0	0	0	0	0	0	0
	C6	0	2	0	0	0	150	0	6	0	0	0	0	0	0
	C7	0	0	11	0	1	0	72	0	0	0	0	0	0	0
	C8	0	0	0	0	0	0	0	34	0	0	0	0	0	0
	C9	0	0	0	0	0	0	0	0	44	0	1	0	0	0
	C10	0	0	0	0	0	0	0	0	0	14	0	0	0	0
	C11	0	0	0	0	0	0	0	0	0	0	19	0	0	0
	C12	0	0	0	0	0	0	0	0	0	0	0	10	0	0
	C13	0	0	0	0	0	0	0	0	0	0	0	0	10	0
	C14	0	0	2	0	0	0	0	0	0	0	0	0	0	9

Table S7. Error matrix of BgPCA-NMI for Washington DC Mall dataset.

		Predicted Class					
		**C1**	**C2**	**C3**	**C4**	**C5**	**C6**
**Original Class**	C1	16	0	0	2	0	0
	C2	0	1032	0	0	0	2
	C3	0	0	108	0	0	0
	C4	0	0	0	474	0	0
	C5	0	9	0	0	369	0
	C6	0	15	0	0	0	848
